# Scatalogic Rites of All Nations

**Published:** 1892-03

**Authors:** 


*Scatalogic Rites of All Nations.* By Captain JOHN G. BOURKE. Pp. 496. Washington: W. H. Lowdermilk & Co. 1891.

Many vile deeds have been done in the name of religion; in all ages and in every form of worship the cult of filth has somehow crept in. The curious in this unsavoury department of knowledge will, in this work, find an extraordinary collection of facts, gathered from every possible source, which have any bearing directly or indirectly on the ritual worship of pure filth. The pure philosopher may trace the gradations between an existence in sackcloth and ashes, and the eating of human excrement; the medical man will probably class the habits as outcomes of aberrations of intellect begotten of excessive or ill-directed zeal in a cause which erring human nature has much at heart.

